# Limited efficacy of zonisamide in the treatment of refractory infantile spasms

**DOI:** 10.1002/epi4.12381

**Published:** 2020-01-24

**Authors:** Shaun A. Hussain, Mario Navarro, Jaeden Heesch, Matthew Ji, Brenda Asilnejad, Haley Peters, Rajsekar R. Rajaraman, Raman Sankar

**Affiliations:** ^1^ Department of Pediatrics (Division of Pediatric Neurology) David Geffen School of Medicine and UCLA Mattel Children’s Hospital Los Angeles California; ^2^ Children’s Discovery and Innovation Institute David Geffen School of Medicine and UCLA Mattel Children’s Hospital Los Angeles California; ^3^ Department of Neurology David Geffen School of Medicine and UCLA Mattel Children’s Hospital Los Angeles California

**Keywords:** epileptic spasms, hypsarrhythmia, West syndrome

## Abstract

A series of relatively small studies collectively suggest that zonisamide may be effective in the treatment of infantile spasms. Using a large single‐center cohort of children with infantile spasms, we set out to evaluate the efficacy and safety of zonisamide. We retrospectively identified all patients with infantile spasms who were treated with zonisamide at our center. For each patient, we recorded dates of birth, infantile spasms onset, response (if any), and most recent follow‐up. To quantify zonisamide exposure, we recorded daily dosage and patient weight at each sequential encounter so as to allow calculation of peak and weighted‐average weight‐based dosage. We identified 87 children who were treated with zonisamide, of whom 78 had previously been treated with hormonal therapy or vigabatrin. Peak and weighted‐average zonisamide dosage were 7.1 (interquartile range 3.6, 10.2) and 5.4 (interquartile range 3.0, 8.9) mg/kg/day, respectively. Whereas five (6%) patients exhibited resolution of epileptic spasms, only two (2%) patients exhibited video‐EEG confirmed resolution of both epileptic spasms and hypsarrhythmia (electroclinical response). Importantly, both electroclinical responders had not previously been treated with hormonal therapy or vigabatrin; in contrast, none of the 78 children with prior failure of hormonal therapy or vigabatrin subsequently responded to zonisamide. Zonisamide was well tolerated, and there were no deaths. This study suggests that zonisamide exhibits favorable tolerability but very limited efficacy among patients who do not respond to first‐line therapy.

## 1 INTRODUCTION

Infantile spasms (IS) is a frequently devastating form of epileptic encephalopathy that usually presents in the first year of life with epileptic spasms, hypsarrhythmia (including variations thereof), and neurodevelopmental arrest.^1^ Unsuccessful treatment is linked to adverse long‐term developmental outcomes.^2^ There is relatively broad consensus that the most effective first‐line therapies for IS are vigabatrin and an array of hormonal therapies,^3^, ^4^ including oral corticosteroids and intramuscular adrenocorticotropic hormone (ACTH). Given that (i) the cumulative short‐term rate of response to hormonal therapy and vigabatrin, administered simultaneously^5^ or sequentially,^6^, ^7^ is approximately 66%, and (ii) the cumulative risk of IS relapse among children followed to age 4 years approaches 50%,^8^, ^9^ we estimate that the first‐line therapies yield sustained long‐term remission in just one‐third of patients with IS. Children with refractory IS have few compelling therapeutic options, aside from epilepsy surgery for the small minority of children with well‐defined focal structural lesions. After failure of first‐line therapy, practitioners and parents must choose from an array of therapies supported by limited and often conflicting data, including zonisamide, topiramate, valproic acid, felbamate, various benzodiazepines, cannabidiol, the ketogenic diet, and corpus callosotomy.^10^ At our center, we have frequently chosen zonisamide as a treatment for refractory patients, on the basis of generally favorable—albeit methodologically limited—reports suggesting that it is both safe and effective.^11^, ^12^, ^13^, ^14^, ^15^, ^16^, ^17^ In this study, we leveraged a large patient cohort to retrospectively evaluate the efficacy and tolerability of zonisamide in the treatment of IS.

## 2 METHODS

### 2.1 Standard protocol approvals

This study was approved by the institutional review board at the University of California, Los Angeles. As this was a retrospective cohort study, the requirement for written informed consent was waived.

### 2.2 Study design

This was a retrospective cohort study. Using a database which includes all patients who underwent video‐EEG at the UCLA Mattel Children's Hospital, we identified patients with video‐EEG confirmed epileptic spasms who were treated with zonisamide. There were no other inclusion or exclusion criteria.

### 2.3 Data acquisition

All data were abstracted from the electronic medical record. For each patient, we recorded the dates of (1) birth, (2) onset of epileptic spasms, (3) initiation of zonisamide, (4) response (if any), and (5) most recent follow‐up. To quantify zonisamide exposure, we reviewed all pediatric neurology progress notes and recorded patients’ weight (kg), and total daily dosage (mg/day) at each encounter to facilitate calculation of the peak weight‐based dose (mg/kg/day) and weighted‐average weight‐based dose (mg/kg/day). As we were chiefly interested in prompt response to zonisamide, we only evaluated the first three months of zonisamide therapy, with the assumption that response after 90 days is unlikely to be attributed to zonisamide.

### 2.4 Efficacy outcome measures

Clinical response was defined as parent‐reported resolution of epileptic spasms, within 3 months of initiating zonisamide, without return of epileptic spasms over the next month. Electroclinical response was defined as clinical response (as defined above) accompanied by overnight video‐EEG‐confirmed absence of epileptic spasms and hypsarrhythmia.

### 2.5 Adverse events

With regard to treatment‐emergent adverse events during zonisamide therapy, we focused on sedation, appetite reduction and/or weight loss, acidosis (serum HCO_3_− <20 mmol/L), nephrolithiasis, and oligohydrosis noted in the medical record. Each of these potential side effects was deemed present or absent; we did not evaluate the severity of any adverse event.

### 2.6 Statistical methods

Summary statistics for continuous variables were presented as median and interquartile range (IQR) given nonparametric distributions. Comparisons of continuous and dichotomous variables were accomplished with the Wilcoxon rank‐sum test and the Fisher exact test, respectively. Ninety‐five percent confidence intervals (95% CI) for small percentages were derived using the Poisson distribution. All comparisons were two‐sided, and only *P* values less than .05 were considered statistically significant. Statistical calculations were facilitated with STATA software (Statacorp, version 14, College Station, Texas, USA).

## 3 RESULTS

### 3.1 Subjects

Baseline clinical and demographic characteristics of the study population are presented in the Table 1. From a cohort of 474 patients with video‐EEG confirmed infantile spasms who were evaluated at UCLA between January 2008 and January 2018, we identified 87 (35 female) children who were treated with zonisamide. With regard to etiology, 58 (67%) patients exhibited known etiology and the most common specific etiologies were hypoxic ischemic encephalopathy/stroke (n = 11), focal cortical dysplasia (n = 10), tuberous sclerosis complex (n = 4), Down syndrome (n = 4), and lissencephaly (n = 4). Of note, median age at zonisamide initiation was 17.4 months (9.8, 35.9) and median latency from epileptic spasms onset to zonisamide treatment was 9.1 months (1.7, 28.7). Antecedent first‐line therapy (ACTH, corticosteroids, or vigabatrin) was observed among 78 (90%) patients, and the median number of treatment failures prior to zonisamide exposure was 3 (IQR 1, 5).

**Table 1:** Baseline characteristics of the study population

Total patients, n	87
Female, n (%)	35 (40%)
Age of onset of IS, months, median (IQR)	6.0 (4.0, 11.9)
Latency from IS onset to ZNS initiation, months, median (IQR)	9.1 (1.7, 28.7)
Hypsarrhythmia present at protocol entry, n (%)	30 (48%)
Number of treatments prior to ZNS, median (IQR)	3 (1, 5)
Prior treatment with hormonal therapy and/or vigabatrin, n (%)	78 (90%)
Development	
Normal development prior to onset of IS, n (%)	43 (49%)
Etiology	
Unknown, n (%)	29 (33%)
Known^a^, n (%)	58 (67%)
Structural, n (%)	43 (49%)
Genetic, n (%)	27 (31%)
Metabolic, n (%)	3 (3%)
Total duration of follow‐up, mo, median (IQR)	55.7 (21.2, 83.0)

Abbreviations: IS, infantile spasms; IQR, interquartile range; ZNS, zonisamide.

a The sum of specific etiologic classifications (ie, structural, genetic, metabolic) exceeded the sum of patients with known etiology because some patients exhibited dual classification.

### 3.2 Zonisamide exposure and efficacy

Median peak and weighted‐average weight‐based zonisamide dosage were 7.1 mg/kg/day (IQR 3.6, 10.2) and 5.4 mg/kg/day (IQR 3.0, 8.9), respectively. Five (6%) children were classified as clinical responders. Of these five, only two patients underwent video‐EEG to confirm response, and both were classified as electroclinical responders. Neither electroclinical responder was previously treated with hormonal therapy or vigabatrin.

The first electroclinical responder presented with a three‐week history of infantile spasms at age 9 months (corrected age 6 months) following a premature birth at 27‐week gestation that was complicated by hypoxic ischemic encephalopathy, as well as necrotizing enterocolitis leading to short‐gut syndrome and dependence on total parental nutrition with the presence of a chronic indwelling central venous catheter. Although the interictal EEG at presentation did not fulfill strict voltage criteria for hypsarrhythmia, it did exhibit “moderately high voltage,” diffuse slowing, disorganization, discontinuity (frequent interictal generalized voltage attenuation), and frequent multifocal epileptiform discharges. The patient was deemed “medically fragile” and treated first‐line with zonisamide. Clinical response accompanied rapid titration to a dose of 9 mg/kg/day over three weeks, and follow‐up overnight video‐EEG confirmed resolution of epileptic spasms and improvement of the interictal EEG (normalization of voltage and discontinuity, improvement in organization, but persistence of diffuse slowing and frequent epileptiform discharges). At most recent follow‐up (age 9 years), the patient maintained seizure freedom, albeit with severe global developmental impairment.

The second electroclinical responder presented with a two‐week history of infantile spasms at age 7 months, with history significant for asymmetric rolling and early hand preference, but otherwise normal development. EEG at presentation demonstrated hypsarrhythmia. The patient was treated first‐line with zonisamide (3 mg/kg/day), though the rationale for selecting zonisamide was not articulated in the medical record. Regardless, zonisamide initiation accompanied immediate clinical resolution (and precluded further anticipated dose titration) and follow‐up overnight video‐EEG confirmed resolution of both epileptic spasms and hypsarrhythmia, and demonstrated the emergence of focal slowing. Subsequent MRI brain identified focal cortical dysplasia. The patient was treated with zonisamide for 1 year, without any other medical or surgical treatment. At most recent follow‐up at age 40 months, the patient is seizure‐free and developing normally.

Among the three patients with clinical response lacking video‐EEG confirmation, we found that one patient promptly transitioned to Lennox‐Gastaut syndrome, and in the other two cases, we suspect response is better attributed to hormonal therapy prescribed less than one month following zonisamide initiation. Nevertheless, we report an overall clinical response rate of 5/87 (6%) and electroclinical response rate of 2/87 (2%). Among the 78 refractory patients (defined as having failed hormonal therapy or vigabatrin), the electroclinical response rate was 0% (95% CI: 0, 4%). Among the 9 patients treated with zonisamide prior to hormonal therapy or vigabatrin, the electroclinical response rate was 22% (95% CI: 3, 80%).

### 3.3 Safety and tolerability

Although zonisamide was generally well tolerated, adverse events were common in this cohort. In our targeted search for side effects noted in the medical record, we encountered sedation in 9 (10%) patients and loss of appetite and/or weight loss in 15 (17%). Twenty‐seven (31%) patients had at least one serum chemistry obtained, and metabolic acidosis was documented in 13 (15%) cases. There were no identified cases of nephrolithiasis or oligohydrosis. There were no deaths, and we did not encounter any cases in which a side effect was identified as a specific reason for zonisamide discontinuation.

## 4 DISCUSSION

**Figure 1:**
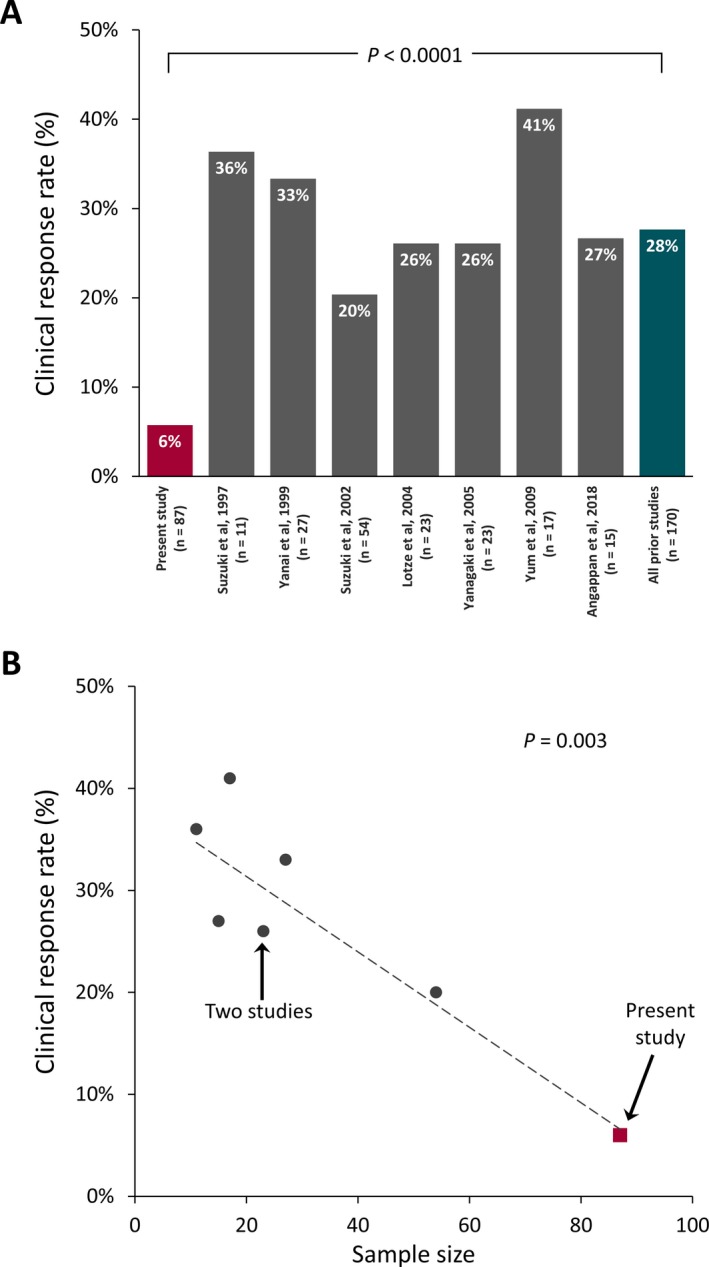
Comparison of zonisamide efficacy across studies. A, Response rate in the present study was lower than all previous reports. B, Across all studies, response rate was inversely proportional to sample size, suggesting potential selection and publication biases. The dashed line represents the least‐squares regression line

This study is noteworthy in that we have reported low rates of clinical and electroclinical response to zonisamide among a large cohort of children with highly refractory infantile spasms. The patients included in our cohort typically exhibited multiple months of infantile spasms and numerous treatment failures prior to zonisamide initiation. Importantly, as there were only nine patients treated with zonisamide prior to hormonal therapy or vigabatrin, we cannot provide a precise estimate of efficacy among nonrefractory patients. Our modest estimate of efficacy contrasts with prior studies which all reported substantially higher response rates (Figure 1, Panel A). Moreover, our very low estimate of clinical response rate is likely an overestimate, as we included a patient with prompt transition to Lennox‐Gastaut syndrome and two patients for whom response may be better attributed to other therapies. There are several potential explanations for this discrepancy. First, we suspect multiple potential selection biases: Our response rate may be especially low because we seldom administered zonisamide prior to hormonal therapy and vigabatrin, thus targeting a refractory cohort. Indeed, both electroclinical responders in this study were among the 9 patients who did not receive hormonal therapy or vigabatrin before zonisamide. Similarly, zonisamide was utilized late in the course of disease, with long intervals between infantile spasms onset and zonisamide initiation, and typically following several treatment failures. Conversely, prior studies may have overestimated response rates. In considering all prior reports, we note that response rate was inversely correlated with sample size (Figure 1, Panel B; *R* ^2^ = .79, *P* = .003), thereby suggesting either small, highly selected cohorts, or potentially some degree of publication bias. For example, it is plausible that investigators who previously observed low response to zonisamide among very small cohorts were not motivated to publish their negative findings. Aside from possible selection and publication biases, variation in dosage may have impacted response rates. Although the peak and weighted‐average dosages observed in this study were comparable to most prior studies, they were far lower than the 30 mg/kg/day dosage used by Yum and colleagues.^16^ Nevertheless, it is possible that our response rates would have been higher if we had implemented either higher dosage or more rapid dose titration. More generally, it should be emphasized that neither this study nor any of the previous studies are randomized, double‐blind, placebo‐controlled studies; all are vulnerable to potential biases in the parent report of epileptic spasms resolution, as well as biases that impact the identification of hypsarrhythmia (and resolution thereof).^18^

With respect to safety and tolerability, this study supports the impression that zonisamide is generally safe and well‐tolerated, especially relative to hormonal therapy and vigabatrin. However, we acknowledge that our approach to identify and quantify adverse events was limited. It is probable that some side effects were poorly documented in the medical record and that we have underestimated adverse event rates as a consequence.

Given the broad consensus that hormonal therapy and/or vigabatrin should be used first‐line for treatment of infantile spasms,^3^, ^4^, ^6^ this study provides little support for the use of zonisamide after failure of hormonal therapy and vigabatrin, especially after long durations of epilepsy. This is consistent with the findings of a contemporary prospective observational study conducted by the (United States) National Infantile Spasms Consortium which found that responses to “nonstandard” therapies (including zonisamide) were inferior to hormonal therapy and vigabatrin.^6^ Similarly, our results echo the findings of a similarly designed study^19^ which found that the efficacy of topiramate (also a carbonic anhydrase inhibitor) for treatment of infantile spasms is very low, especially in comparison with earlier studies which had reported high rates of response.^20^, ^21^ To the extent that these results conflict with prior studies suggesting more robust efficacy, we welcome future efforts to evaluate zonisamide for treatment of infantile spasms, ideally targeting large cohorts of children with less refractory disease (eg, shorter duration of epilepsy), with implementation of more rigorous experimental designs.

## CONFLICTS OF INTEREST

Dr Hussain has received research support from the Epilepsy Therapy Project, the Milken Family Foundation, the Hughes Family Foundation, the Elsie and Isaac Fogelman Endowment, Eisai, Lundbeck, Insys, GW Pharma, UCB Biopharma, Zogenix, and the NIH (R34MH089299). He has received honoraria for service on the scientific advisory boards of Mallinckrodt, Upsher‐Smith Laboratories, Insys, UCB Biopharma, and Zogenix; as a consultant to UCB, Mallinckrodt, Insys, GW Pharma, West Therapeutic Development, Aquestive Therapeutics, Shennox, and Amzell; and on the speaker's bureau for Mallinckrodt and GW Pharmaceuticals. Dr Rajaraman has received research support from Marinus and the International CDKL5 Research Foundation. Dr Sankar serves on scientific advisory boards or serves on the speaker's bureau for and has received honoraria and funding for travel from Eisai, UCB Pharma, Sunovion, Supernus, Greenwich Biosciences, LivaNova, and on the advisory boards for Aquestive Therapeutics, West Therapeutic Development, Insys Development Company, and Zogenix; receives royalties from the publication of *Pellock's Pediatric Neurology, 4th ed.* (Demos Publishing, 20172008) and *Epilepsy: Mechanisms, Models, and Translational Perspectives* (CRC Press, 2011); serves on speakers’ bureaus for and has received speaker honoraria from Eisai, UCB, GlaxoSmithKline, LivaNova, Supernus, and BioMarin. He has received research support from SK Life Sciences and Insys Development Company, Inc The remaining authors report no conflicts of interest. We confirm that we have read the Journal's position on issues involved in ethical publication and affirm that this report is consistent with those guidelines.
